# Pseudoaneurysm of the Profunda Femoris Artery following Blunt Trauma Treated by Endovascular Coil Embolization: Review of Two Cases and Relevant Literature

**DOI:** 10.1155/2017/8079674

**Published:** 2017-01-26

**Authors:** Saptarshi Biswas, Patrick McNerney, Paul Kiproff

**Affiliations:** ^1^Department of Trauma and Acute Care Surgery, Forbes Regional Hospital, Allegheny Health Network, Monroeville, PA, USA; ^2^Department of Interventional Radiology, Forbes Regional Hospital, Allegheny Health Network, Monroeville, PA, USA

## Abstract

Profunda femoris artery (PFA) pseudoaneurysm after blunt trauma without associated femur fracture is a rare occurrence. Most of the reported cases of PFA pseudoaneurysm in the English literature developed after penetrating trauma, surgical procedures, and femur fractures. We present two such cases following blunt trauma and without any associated long bone injury. After initial imaging failed to show any long bone fracture, CT angiography confirmed pseudoaneurysm of the branch of the PFA. Both patients were then treated with emergent coil embolization of the bleeding vessel. Pseudoaneurysms typically present late and signs of persistent hip pain, thigh swelling, presence of a pulsatile mass, and even unexplained anemia all may suggest the diagnosis. Recognition of PFA pseudoaneurysm requires high index of suspicion and is often difficult to diagnose clinically because of its location.

## 1. Introduction

Injury to the PFA is not an uncommon occurrence, often resulting from fractures of the nearby femur bone or penetrating trauma [1]. Iatrogenic sources, generally occurring during or resulting from surgery, are also a possible cause of PFA injury [2]. Injuries to the PFA precipitated by the aforementioned sources may result in pseudoaneurysm [2]. However, PFA pseudoaneurysms resulting from blunt trauma are rather atypical especially with no associated surgery or long bone fracture [3].

Pseudoaneurysms of the PFA will likely present with a novel pulsatile and painful mass; however, pulsations may be masked by either hematoma or thrombus formation potentially complicating clinical diagnosis [4]. Masking of symptoms and abnormal etiology, such as blunt trauma, can complicate the diagnosis of PFA pseudoaneurysm [4]. A high index of suspicion must be maintained by physicians in these situations to prevent complications associated with late stage diagnosis.

We report two cases of a profunda femoris pseudoaneurysm occurring secondary to a blunt trauma involving no long bone fractures or penetrating injury, subsequently confirmed by CT angiography, and later successful treatment via coil embolization.

## 2. Case Report

*Case 1.* A 47-year-old male presented to the emergency department after falling backwards while trying to put his motorcycle in the rear of his pickup truck. He sustained an injury to his left hip and thigh. The patient was initially capable of weight bearing but as time progressed he experienced severe swelling and pain in the region that radiated to his groin. The swelling in the left thigh progressively worsened during the patient's time in the ED; however, no neurological or vascular compromise of the distal left lower extremity was noted. Past medical history was significant for bipolar 1 disorder and left foot fracture. Surgical history was positive for an inguinal hernia repair. The patient's current medications were quetiapine fumarate and NSAIDs (nonsteroidal anti-inflammatory drugs). Vitals and labs at presentation to the ED were essentially within normal limits. His hemoglobin was 12.7 and hematocrit was 36.6.

**Figure 1:**
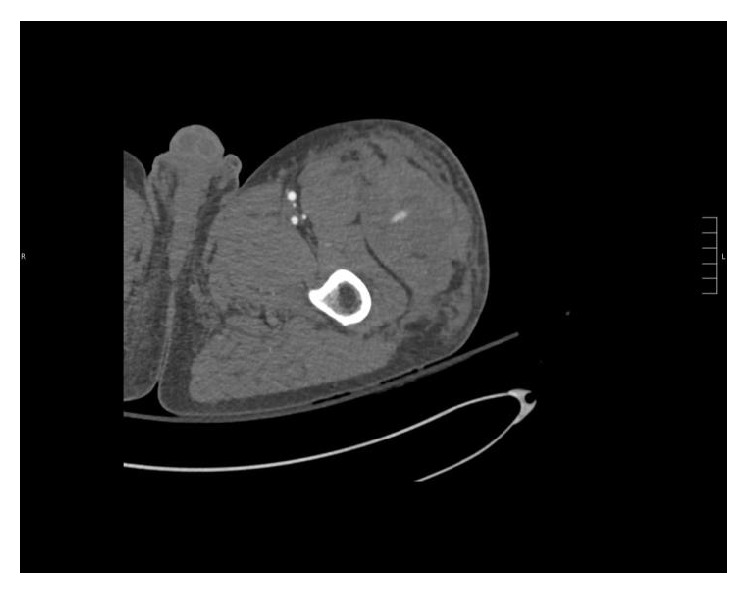
Contrasted enhanced CT of the left thigh demonstrates an area of extravasation and surrounding hematoma.

**Figure 2:**
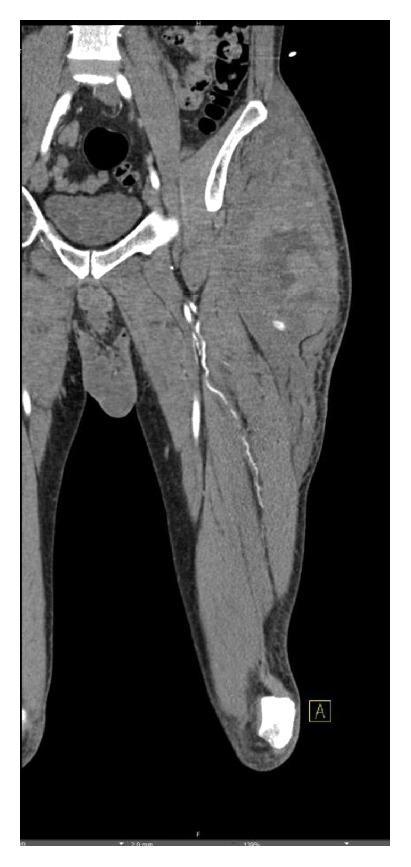
Coronal CT reconstruction further details the area of extravasation and associated hematoma.

Imaging initially included a chest and pelvic X-ray which showed no gross abnormality. A CT angiogram of the left lower extremity was performed for increasing swelling which revealed a large soft tissue hematoma along the left hemipelvis and upper thigh with active extravasation likely emanating from a small branch of the left profunda femoris (focus of contrast blush) (Figures 1 and 2).

**Figure 3:**
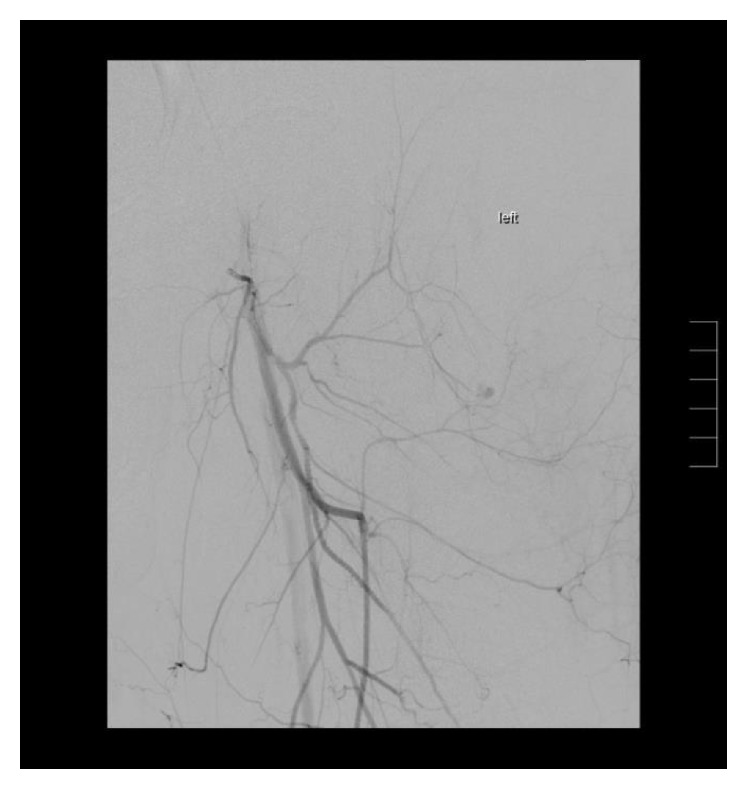
Left lower extremity angiogram demonstrates an area of contrast extravasation arising from a peripheral deep femoral branch.

**Figure 4:**
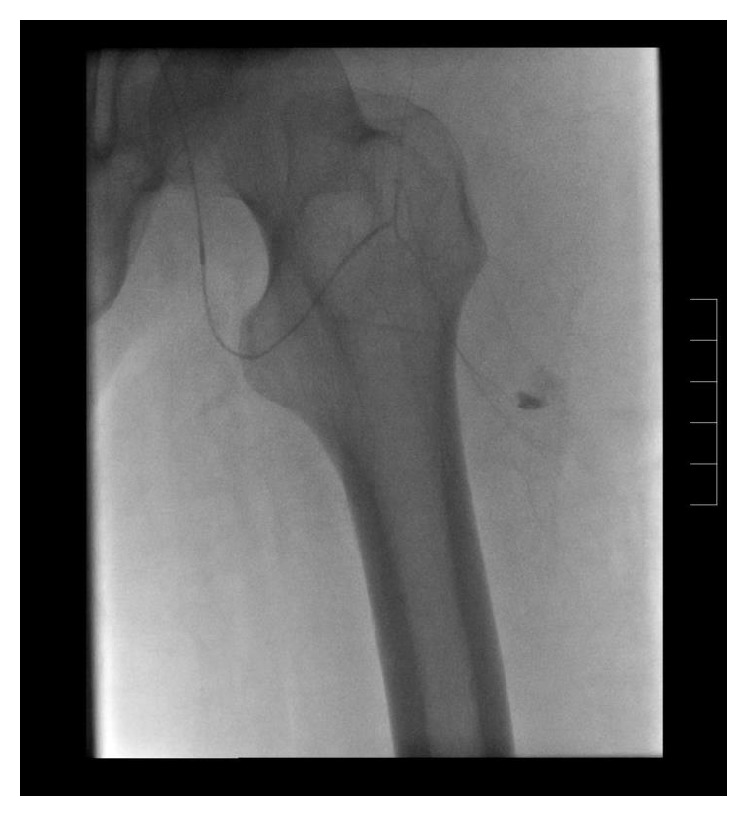
Selective catheterization confirmed the supply to the bleeding vessel.

**Figure 5:**
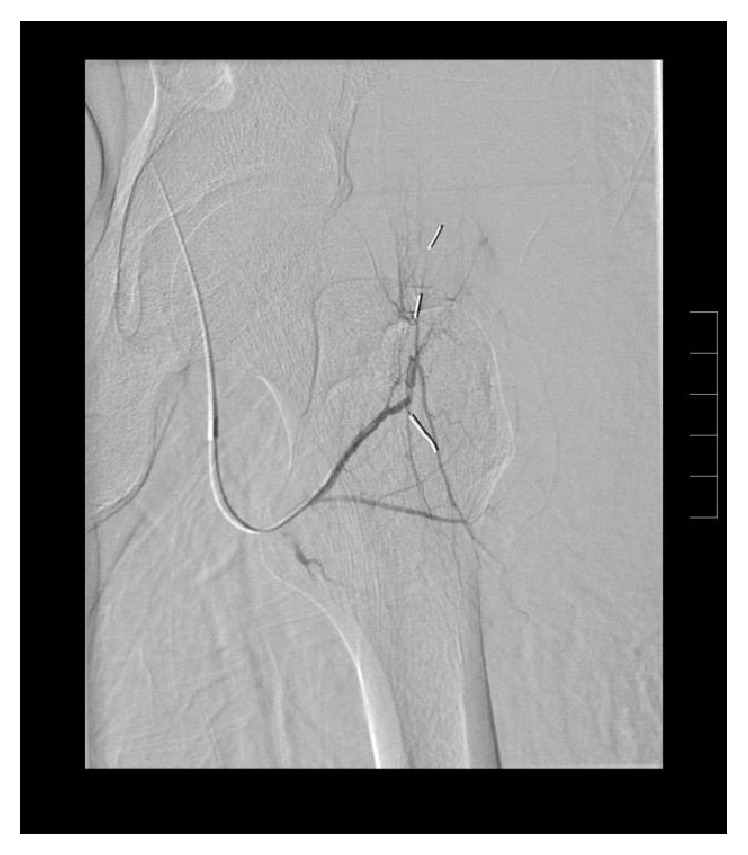
Postembolization angiogram confirms that hemostasis has been achieved.

The patient was subsequently treated by endovascular means. The right common femoral artery access was used with a micropuncture technique under ultrasound guidance. A standard guidewire was then placed followed by a 5-French sheath. Following this, an Omni flush catheter and LLT guidewire were used to traverse the aortic bifurcation and position the catheter within the left external iliac artery. Digital angiography was performed and a site of extravasation was noted within a small peripheral branch of the profunda (Figures 3 and 4). The Omni flush was then exchanged for a 4-French straight catheter that was positioned within the proximal left profunda. Angiography was performed and, perplexingly, no active extravasation was noted. A Progreat microcatheter was then introduced in a coaxial fashion into the first perforating artery. Repeat angiography identified extravasation arising from a left distal profunda branch artery. The vessel proximal and distal to the area of active bleeding was then embolized using three 0.5 mm straight hilar microcoils. Repeat angiography demonstrated successful embolization and no further extravasation (Figure 5).

All catheters were removed and hemostasis was secured. The patient was discharged home on oral analgesics on hospital day 3.

*Case 2.* A 65-year-old female presented to the ED via emergency helicopter transport after being struck by a vehicle as a pedestrian while attempting to enter her own vehicle. The patient stated that she was struck by the vehicle on the right side and then fell down on her knees; she denied head trauma and loss of consciousness. Her past medical history was positive for arthritis and gastroesophageal reflux disease. She denied past surgical history and stated that she does not regularly take any medications.

Primary survey was essentially normal; secondary survey revealed right rib pain, right thigh pain, and lower back pain. Vitals were within normal limits. Labs showed WBC of 12.91, hemoglobin of 11.2, and hematocrit of 34.0.

**Figure 6:**
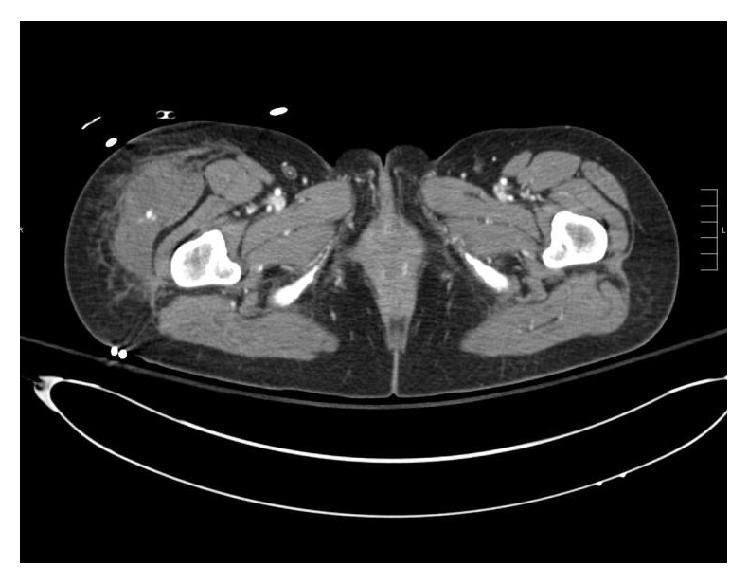
Contrast enhanced CT demonstrates an area of active extravasation and surrounding hematoma.

**Figure 7:**
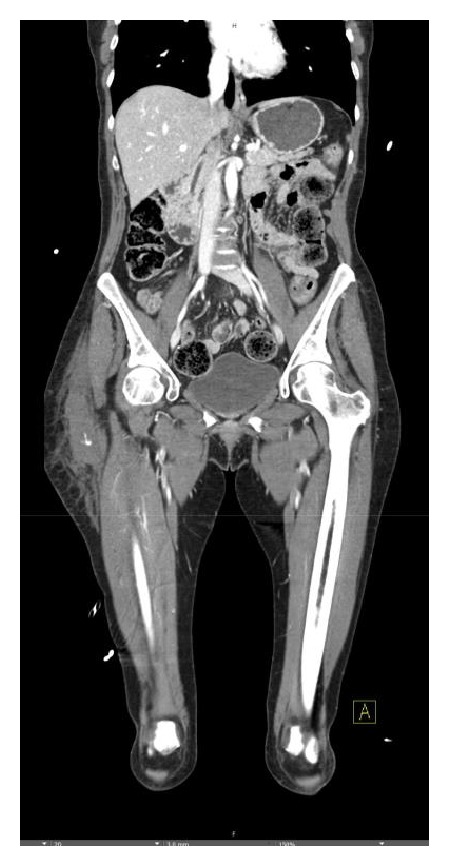
Coronal CT reconstructed images further detail the area of active extravasation and surrounding hematoma.

CT of the face and head was also negative for injury. Chest CT showed a small right hemopneumothorax with displaced fractures of the right 9th through 12th ribs as well as a mild pulmonary contusion of the right lower lobe. CT of the thoracolumbar and cervical spine showed no vertebral fractures. X-rays of the pelvis and knee showed only soft tissue swelling. Abdomen/pelvis CT demonstrated a moderate size hematoma that was overlying the right hip with a 5 mm contrast blush suggesting active bleeding (Figures 6 and 7).

**Figure 8:**
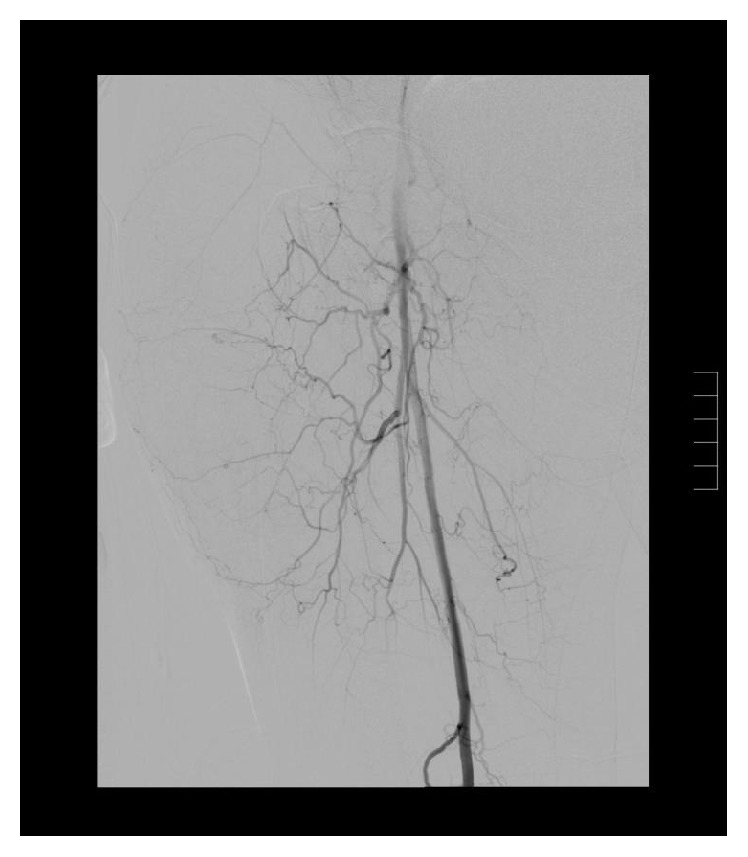
Right lower extremity angiogram demonstrates vague area of extravasation.

**Figure 9:**
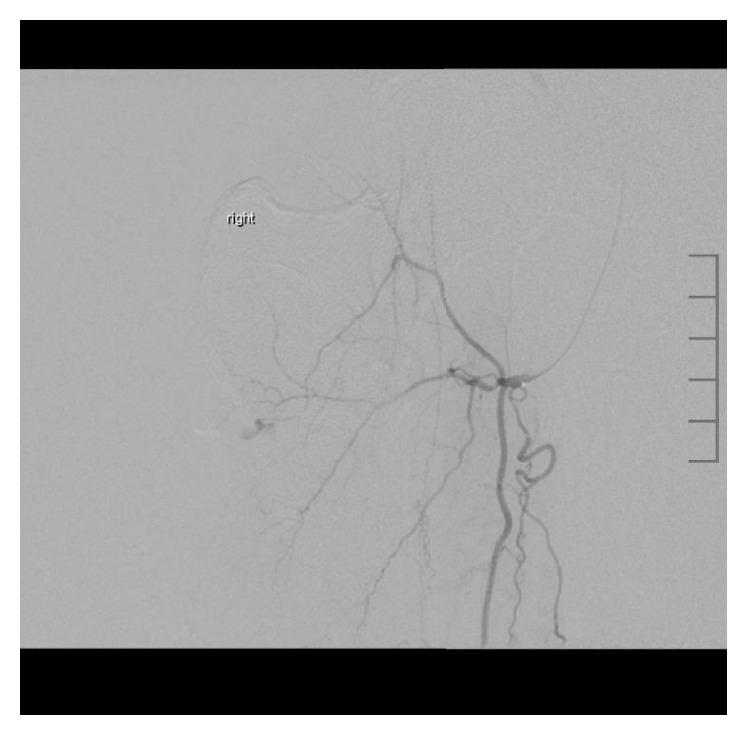
Selective deep femoral branch arteriogram further details the area of active bleeding.

**Figure 10:**
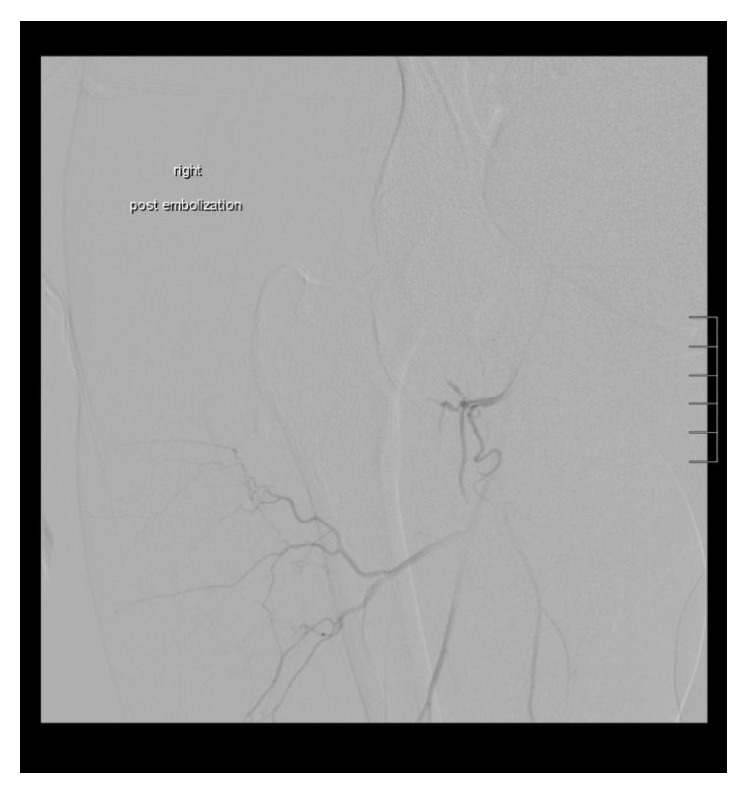
Postembolization arteriogram confirms successful hemostasis.

Interventional radiology was consulted and the patient underwent pelvic arteriogram. From the contralateral approach, the left common femoral artery was accessed and a 5-French sheath was placed. A 4-French Omni flush catheter was then advanced over the guidewire to the aortic bifurcation. Position was confirmed with a small hand contrast injection and no stenosis or extravasation was noted. A 0.035-inch Glidewire was then advanced through the 4-French Omni flush catheter, which was subsequently removed and replaced by a 5-French straight multi-side-hole flush catheter. The 5-French catheter was then positioned within the right external iliac artery and an area of active arterial extravasation and pseudoaneurysm formation was noted to be originating from the subsegmental transverse branch of lateral circumflex femoral branch of the right profunda femoris artery (Figures 8 and 9). A 2.8-French Progreat microcatheter was then advanced in a coaxial manner through the 5-French base catheter allowing for the use of a 0.016-inch primary micro-Glidewire and a 0.016-inch GT 2 Glidewire to catheterize the medial and lateral circumflex femoral branch of the right profunda femoris. Angiogram was performed in both branches and again demonstrated the pseudoaneurysm formation within the subsegmental transverse branch of lateral circumflex femoral branch which was then selectively embolized by Gelfoam slurry. No further active arterial extravasation was noted after repeat angiogram (Figure 10), all catheters were withdrawn, and a 5F Mynx vascular closure device was deployed.

The patient tolerated the procedure well and was eventually discharged 4 days later following a significant improvement in her condition. The patients were doing well in their first postdischarge visits in the trauma clinic one week later. Doppler USG on bilateral lower extremities was performed during the postdischarge first follow-up clinic visit. However, they were lost to subsequent follow-ups.

## 3. Discussion

Comprehension of the anatomy of the upper part of lower extremity is essential in understanding the various complications that can result in a profunda femoris pseudoaneurysm. The femoral artery courses into the leg through the femoral triangle before splitting into the superficial femoral artery and the profunda femoris [5]. The superficial femoral artery goes on to supply 5 branches: the superficial circumflex iliac, superficial epigastric, superficial external pudendal, deep external pudendal, and descending genicular artery [5, 6]. The profunda femoris then terminates within the thigh, never leaving the region [5]. Several branches of the profunda femoris exist but the clinically significant ones are the medial and lateral femoral circumflex arteries. The medial and lateral femoral circumflex arteries supply the head of the femur [5]. The medial femoral circumflex artery injury is well known for being a possible complication following a femoral neck fracture, potentially leading to avascular necrosis of the femoral head and neck [5]. Other notable, though less clinically relevant, branches of the profunda femoris are the perforating arteries, which typically are 3 to 4 in number and branch off the profunda femoris in a pattern based on their numerical order [7].

Several fairly common aberrations exist concerning the profunda femoris and its branches. Occasionally, the number of branches of the profunda femoris differs; there may be three or four perforating arteries and both the lateral and the medial femoral circumflex arteries may arise directly from the femoral artery [8]. The lateral femoral circumflex may also course posterior to the femoral nerve rather than through it [9]. More rare occurrences have been described such as an aberrant tortuous vessel described by Singh et al. that was discovered during exploration of what was thought to be a profunda femoris pseudoaneurysm but was found to be a pseudoaneurysm of the aberrant vessel [10].

A pseudoaneurysm, also known as a false aneurysm, is a hematoma not contained by vessel walls but rather contained by a thin fibrous capsule [11]. The mass is usually pulsatile in nature, which can aid in diagnosis. For the pseudoaneurysm to persist, it must have a communication with the originating artery [11]. Pseudoaneurysms typically will result from penetrating or blunt trauma, especially if it involves bone fractures, as well as surgical or procedural interventions [11]. Infection and severe inflammation have also been known to be a source of pseudoaneurysm [11]. Pseudoaneurysms are also much more common in those currently undergoing anticoagulation therapy [11].

The potential causes of profunda femoris pseudoaneurysms mirror those of any pseudoaneurysm: iatrogenic cause, trauma (blunt or penetrating), long bone fracture, and potentially inflammation or infection [1, 11]. Pseudoaneurysms, occurring as a result of orthopedic surgery, are well documented. Damage to the PFA during surgery capable of inciting pseudoaneurysm formation can result from bone spikes, screw tips, drills, displaced implants, retractors, and of course the lancet itself [12]. A case reported by Unay et al. discussed the formation of a PFA pseudoaneurysm after surgical internal fixation of a left subtrochanteric femur fracture [12]. The pseudoaneurysm was suspected to be caused not by the fracture but rather by the bone spikes; this was determined due to the distance of the PFA injury from the fracture and surgical site [12]. Another case of iatrogenic PFA pseudoaneurysm was described by Karmakar and Horsley during a total hip arthroplasty in which the sharp Hohmann retractor was likely the cause of the injury [13]. The authors suggested an alternate hypothesis in which excessive retraction was the cause of the injury [13]. Use of a blunt Hohmann retractor was recommended by the authors as a result [13]. Another incident in the literature concerning PFA pseudoaneurysm following total hip arthroplasty, described by Huynh et al., suggested that inappropriate use of the Hohmann retractor was again the source of the injury [14]. Harper et al. suggested a more complex etiology of PFA injury during hip arthroplasty possibly resulting from a stretch injury to the vasculature of the region [6]. This etiology of injury would be similar to lateral femoral cutaneous nerve palsies that have been known to occasionally result from stretch injuries during hip arthroplasty positioning [6].

Penetrating trauma and long bone fracture are also a common source of PFA pseudoaneurysm [1]. Penetrating structures and bone fragments can easily injure the PFA requiring emergency physicians and trauma surgeons to have high clinical suspicion for the injury when encountering patients which these mechanisms of injury [1]. Despite the traumatic nature of the PFA pseudoaneurysm, a delayed presentation is not uncommon. The literature describes a case in which a patient presented with claudication and swelling in the region one month after a penetrating injury [15]. The case, described by Besir et al., demonstrates the often lengthy time to presentation [15]. Butterworth et al. described an even more poignant example of this by describing a case in which a patient presented with a PFA pseudoaneurysm 3 years after penetrating injury [2]. One study goes as far to suggest that average time from initiating injury to diagnosis ranges from 4 months to 6 years [6]. Fracture, particularly of the hip, can result in bone shards inducing PFA pseudoaneurysm in a similar manner to penetrating trauma. Two cases in the literature describe situations of intertrochanteric hip fracture resulting in bone shards damaging the PFA. One of such cases resulted from a fall, described by Cowley et al., and the other resulted from what was described as trivial trauma by Sharma et al. [16, 17]. These two cases suggest that physicians and surgeons should have a high index of suspicion for PFA pseudoaneurysm during instances of fracture especially if the said fracture is an intertrochanteric fracture of the hip [16, 17].

PFA pseudoaneurysm following blunt trauma is a rarer source of PFA pseudoaneurysm which is poorly represented within the literature. One situation described was following a rugby injury [3]. Although anterior thigh trauma is common in contact sports, a PFA pseudoaneurysm is a rare complication when fracture is not involved [3]. Young et al. described a case where a young rugby enthusiast was initially thought to have acute compartment syndrome only for the diagnosis of PFA pseudoaneurysm to be made 10 days after the fasciotomy for his compartment syndrome [3]. This case demonstrates the potential difficulty in making a diagnosis when the etiology is not highly suggestive. Despite the diagnosis being confirmed by simple ultrasound, the index of suspicion for pseudoaneurysm was low, due to the nature of the injury being blunt trauma, resulting in a considerably delayed diagnosis [3].

Cases concerning pseudoaneurysm formation involving other vasculatures were found to present with a similar clinical vignette to PFA pseudoaneurysm. Norris et al. described an occasion of pseudoaneurysm following blunt trauma [18]. The patient who presented following blunt trauma to the knee after a motor vehicle collision was initially diagnosed with a hamstring laceration and MCL strain only to return on month later with increased knee swelling and a nonmobile pulsatile mass on the medial side of the knee [18]. The delayed presentation and significant swelling mirror the PFA pseudoaneurysm case and are similar to the previous case that was initially misdiagnosed, in this case being treated with fine needle aspiration of the joint on two occasions before pseudoaneurysm was suspected [18]. After clinical suspicion for pseudoaneurysm was raised, an arteriogram confirmed a superior medial geniculate pseudoaneurysm [18].

Potential complications of a PFA pseudoaneurysm include compression of the femoral vein increasing the possibility of deep vein thrombosis and the complications which can be associated with them [1]. As with any hemorrhage, the risk of anemia and exsanguination also exists with PFA pseudoaneurysms [1]. Hemorrhage into the surrounding tissue also causes the possible complication of acute compartment syndrome [3]. AV fistula formation is also a possibility though less common than those previously mentioned [1].

Diagnosis of PFA pseudoaneurysm is typically initially made based on clinical suspicion. Clinical signs of the condition are often a pulsatile swelling located along the path of the PFA that is often exquisitely painful [12]. Clinical findings can be somewhat concealed depending on location of the bleeding and thrombus formation, complicating diagnosis [4]. Delay in pseudoaneurysm formation, be it hours or potentially months, can also further complicate diagnosis [3]. Once clinical suspicion exists, duplex ultrasonography can be used to confirm the diagnosis with over 90% accuracy [11]. CT angiography is then generally performed for better visualization to aid in treatment planning [4].

Treatment for PFA pseudoaneurysms varies based on a number of factors but is mostly dependent on the size of the pseudoaneurysms [11]. If small enough, some pseudoaneurysms can be simply observed as they will often spontaneously thrombose [11]. IR guided coil embolization is generally the preferred method of treatment for small to moderate size pseudoaneurysms [10]. However, availability of IR facilities is not definite and in situations where embolization is inaccessible surgical intervention is the preferred treatment [10]. Surgery is indicated for large pseudoaneurysms or those incapable of being embolized [11]. Other modalities of treatment that have been described in the literature include US guided obliterative compression and direct thrombin injection [11].

## 4. Conclusion

PFA pseudoaneurysm is not particularly uncommon and is often associated with penetrating trauma, surgical intervention, or femur fracture [2]. PSA pseudoaneurysm is much less commonly associated with blunt trauma [2]. Delayed formation of pseudoaneurysm is common furthering the difficulty in making the diagnosis. Despite its relative rarity, suspicion of PFA pseudoaneurysm must remain high in cases of blunt trauma so as to avoid complications associated with late diagnosis and pseudoaneurysms should not be assumed to be only acute in nature. Complications can include acute blood loss anemia, localized ischemia, compartment syndrome, peripheral occlusive syndrome, and potentially exsanguination if severe enough and delay to treatment is long enough [12]. Once clinical suspicion exists, duplex US is recommended for conformation and then CT angiogram is often performed for planning [11]. Treatment modalities can vary significantly depending on the size of the pseudoaneurysms, with small ones being capable of thrombosing spontaneously and large ones requiring surgery [11]. Pseudoaneurysms that lie in between these two ranges will often be treated with catheter embolization [11].
